# Supplementary material for: A Linkage-Based Genome Assembly for the Mosquito Aedes albopictus and Identification of Chromosomal Regions Affecting Diapause
Source: Insects. 2021 Feb 16;12(2):167. doi: 10.3390/insects12020167 (PMC7919801; doi:10.3390/insects12020167)
Supplement: Supplementary file 1 [file insects-12-00167-s001.pdf]

SUPPLEMENTARY INFORMATION FOR:

A linkage-based genome assembly for the mosquito *Aedes albopictus* and identification of chromosomal regions affecting diapause.

John H. Boyle, Pasi M.A. Rastas, Xin Huang, Austin G. Garner, Indra Vythilingam, Peter A. Armbruster

File S1.1: Intercrossing tropical and temperate lines for linkage mapping and BSA

The intercross lines used for the linkage mapping and BSA were established by mating an individual TROP male and an individual TEMP female for each line. To perform the cross, larvae from laboratory F_3 TROP and F_7 TEMP colonies were reared under near-optimal conditions of larval nutrition, a 16L:8D photoperiod at 21°C, and approximately 80% relative humidity as described previously [24, 25]. Upon pupation, TROP males and TEMP females were placed into separate adult cages. Two days after eclosion, TEMP females were blood fed to repletion on a human host, briefly CO_2 anesthetized, and females with a visible blood bolus were retained.

Sixty mating cages were established with one TROP male and three TEMP females per cage. Each cage consisted of a 1.2-liter inverted bucket with mesh windows and a moist filter paper lining the bottom of the cage. Cages were provisioned with organic raisins (Newman's Own Organic, Aptos, CA.) as a sugar source. After four days to allow for mating, TROP males were snap frozen in liquid nitrogen and stored at -80°C and TEMP females were individually placed into fly vials (28.5×95mm, Genesee Scientific, San Diego, CA, USA) half-filled with deionized water and sealed with mesh and provisioned with an organic raisin. A strip of unbleached paper towel (Seventh Generation, Burlington, VT, USA) was

placed into each fly vial to provide a substrate for oviposition. Two females that oviposited > 20 eggs each were snap frozen in liquid nitrogen and stored at -80°C for subsequent RNAseq genotyping. The eggs (intercross F_1) of these females were maintained and hatched out under the near-optimal conditions as described above to establish two independent intercross lines. Larvae were reared as described above and adults were allowed to random mate in a separate 9.5-liter mass-swarm cage for each line with at least 100 males and 100 females each generation. Both lines were used for the BSA in the intercross F_4 generation. Additionally, one line was arbitrarily chosen and maintained to the intercross F_7 generation to increase recombination among marker SNPs for linkage mapping.

File S1.2: Linkage mapping: Tissue preparation, RNA extraction, and sequencing

In the intercross F_7 generation, larvae were reared to adults as described above. 50 males and 50 females were collected one week after eclosion, snap frozen in liquid nitrogen and individually stored at -80°C. Total RNA was extracted from each of the two parents and 70 individual F_7 intercross mosquitoes using a modified TRI® Reagent (Sigma Aldrich, St. Louis, MO) RNA extraction protocol described in previous publications[17, 19]. Briefly, contaminant DNA was removed from each RNA sample using a Turbo-DNA free kit (Ambion, Austin, Texas). For each sample, RNA integrity and quantity were assessed using an Agilent Bioanalyzer® RNA Chip (Agilent Technologies, Santa Clara, CA, USA). Only samples with an RNA quantity of at least 400 ng and minor or no degradation were retained for library preparation. Samples of total RNA from the two intercross F_0 parents were used for library preparation and sequencing alongside those of other TROP and TEMP

individuals as described below (see SI Section 1.5: " *Bulk segregant analysis: RNA extraction and sequencing of bulks, TEMP, and TROP individuals*"). Samples of total RNA from the 70 individual F_7 offspring were used to create individual sequencing libraries using a modified protocol from the NEBNext® Ultra™ RNA Library Prep Kit and NEBNext Poly(A) mRNA Magnetic Isolation Module (New England Biolabs, Ipswich, Massachusetts). Poly-A mRNA was isolated using Oligo d(T) 25 beads, chemically fragmented for 5 minutes at 94°C, and then converted to cDNA using random primers and a two-step double strand synthesis reaction. Double-stranded cDNA was then purified using an Agencourt AMPure XP bead clean up (Beckman Coulter Genomics, Chaska, MN). 5' overhangs were filled and blunt end double-stranded cDNA was 5' phosphorylated and 3' dA-tailed. Each of the 70 dA-tailed cDNA samples was size selected for ~400bp inserts using Agencourt AMPure XP beads. After size selection, each cDNA sample was individually barcoded with a unique 8-base index using the NEBNext Oligo Adaptor set (96 Singleplex) and PCR enriched in a thermocycler as follows; 1 denaturation step at 98°C for 30 sec, 12 cycles of 98°C for 10 sec and 65°C for 75 sec, and 1 extension step at 65°C for 5 min. Next, cDNA libraries were purified again using an Agencourt AMPure XP bead clean up. Library quality and concentrations were assessed on an Agilent Bioanalyzer® High Sensitivity Chip (Agilent Technologies, Santa Clara, CA, USA). Each library was normalized to 2nM, and 5 pools were made from equal volumes of 14 cDNA libraries for each pool. Libraries were combined into pools based on the composition of barcode sequences according to specifications from the NEBNext Oligo Adapter set (96 Singleplex). Each pool was paired-end sequenced on an individual flow-cell lane of Illumina HiSeq 4000 (read length = 150 bp) at the Institute for Genome Science (IGS), University of Maryland.

File S1.3: Linkage mapping: read cleaning and alignment to the Palatini et al. (2020) assembly

In order to remove contaminant and low-quality reads, all libraries were aligned to the UniVec database of potential DNA vector contaminants [49], as well as *Ae. albopictus* rRNA sequences (Genbank accession L22060.1) and *Ae. albopictus* mitochondrial tRNA sequences (tRNA features from GenBank accession AY072044.1). These potential contaminant sequences were indexed using bowtie2 version 2.2.6 [34]. Alignment of RNAseq reads was performed using bowtie2 version 2.2.6, and any aligned reads were discarded. Scripts for all bioinformatics and analyses are available in our dryad repository at: https://datadryad.org/stash/dataset/doi:10.5061/dryad.mgqnk98z4.

We then used Trimmomatic version 0.39 [50] to trim Illumina adaptor sequences from the reads. We used the dynamictrim tool from SolexaQA++ version 3.1.7.1 [51] to trim off the ends of reads once quality fell below 15 and to remove any read pairs in which either of the pair had a length less than 50 bp. We removed any unpaired reads from each library using custom scripts.

We indexed the Palatini *et al.* (2020) genome assembly (AalbF2), Genbank accession GCA_006496715.1, using the STAR aligner version 2.7.1a [52]. We then performed alignment of sequencing reads from the two intercross F_0 parents and 70 individual F_7 intercross individuals to the AalbF2 assembly using a 2-step alignment process. First, an initial STAR alignment to identify splice junctions in the mRNA libraries, then a second STAR alignment using those splice junctions to inform the alignment. The resulting alignments for each library were further processed using Picard version 2.20.4 [53]to sort

alignments, add read groups, and remove duplicates. We indexed the reference genome using samtools 0.1.19 [27, 54], and created a sequence dictionary using Picard. We then used the SplitNCigarReads tool from the Genome Analysis Toolkit (GATK) version 4.1.2.0 [55] to split alignments of RNA reads at which a splice junction had been identified.

File S1.4: Bulk segregant analysis (BSA): measuring diapause phenotypes

An adult mass-swarm cage was established for each line under a short-day photoperiod; each cage contained approximately 100 females and 100 males. After 11 days under short-day conditions, females were blood fed to repletion as described in SI Section 1.1. Engorged females were then transferred into individual fly vials half-filled with water and provisioned with an organic raisin and unbleached paper towel (Genesee Scientific, San Diego, CA, USA) as described in SI Section 1.1 to stimulate oviposition. Vials were checked daily and individual females were snap frozen in liquid nitrogen and stored at -80°C after they had oviposited at least 20 eggs. Diapause incidence (DI) was measured for eggs collected from individual females maintained under unambiguous short-day photoperiod (SD; 8L:16D) as described previously [25, 56]. Briefly, paper towel strips with eggs were removed from fly vials containing individual females every Monday-Wednesday-Friday (M-W-F), maintained under SD conditions for ~ 48 hr, and then gently air-dried. Egg papers were then stored at approximately 80% relative humidity under SD for at least seven days. Eggs ranging from one to two weeks of age were then stimulated to hatch by submersion in ~75 ml water with ~ 1ml larval food. The number of hatched larvae was recorded and the egg papers were re-dried. This procedure was repeated 7 days later, after which the eggs were bleached [57] to reveal the number of embryonated but unhatched (i.e., diapause)

eggs. DI was calculated as DI = (number of embryonated unhatched eggs)/(number of hatched eggs + number of embryonated unhatched eggs) [25, 56].

File S1.5: Bulk segregant analysis: RNA extraction and sequencing of bulks, TEMP, and TROP individuals

RNA was extracted and sequencing libraries were prepared as described in the manuscript text (see: *"Bulk segregant analysis: RNA extraction and sequencing of bulks, TEMP, and TROP individuals"*). Eleven libraries from the TEMP and TROP individual samples were pooled for paired-end sequencing on one flow-cell lane, and the remaining 9 TEMP and TROP libraries were pooled for paired-end sequencing on another flow-cell lane of an Illumina HiSeq 2000 sequencer (read length = 100 bp). The four bulk libraries were paired-end sequenced on two lanes of an Illumina HiSeq 4000 (read length = 150 bp), with each lane containing the high and low bulks from a single line.

File S1.6: Bulk segregant analysis: SNP calling and filtering

We used HaplotypeCaller to call variants within each individual (or each bulk, adjusting the sample ploidy as appropriate), then used GenomicsDBImport for each scaffold to combine the outputs from HaplotypeCaller from all individuals (and bulks). We then used to GenotypeGVCFs to do joint genotyping on all individuals (and bulks) simultaneously. The large ploidy of the bulks caused genotyping to fail due to lack of memory in some regions of the genome, which were therefore excluded from joint genotyping. These regions represented approximately 50-60 Mb spread across 143 scaffolds. The genotype calls were then combined using GatherVcfs, and a set of high-

quality SNPs output by removing SNPs with quality by depth (QD) < 10, phred-scaled *p*-value of Fisher's Exact Test for strand bias (FS) > 60, root mean square of the mapping quality (MQ) < 35, mapping quality rank sum test (MQRS) < -12.5, read position rank sum test (RPRS) < -8, or strand odds ratio (SOR) > 3.

This set of SNPs was then used as the basis of bootstrap base recalibration using the GATK BaseRecalibrator tool. The alignments produced above were recalibrated using the ApplyBQSR tool, and these alignments used as the basis of a second round of SNP calling, following the protocol outlined above. After base recalibration, approximately 60-70 Mb spread across 148 scaffolds was excluded due to memory failures during joint genotyping. High-quality SNPs were filtered as described above. The AnalyzeCovariates tool showed that a second round of base recalibration based on these SNPs had little effect on the alignment, and so we retained the SNPs identified after a single round of base recalibration. These recalibrated, high-quality SNPs were further filtered to exclude multiallelic loci using the GATK SelectVariants tool. For libraries from single mosquitoes, genotypes with a genotype quality score of 5 or less were removed from the data set.

File S1.7: Bulk segregant analysis: identifying putative diapause-associated SNPs

To calculate a diapause-associated *p*-value based on SNP frequency differences between the TEMP and TROP sample, we calculated the proportion of the reference allele in each sample as the average of its proportion in the TEMP sample and its proportion in the TROP sample. From this, we assigned all SNPs into five categories based on minor allele frequencies (MAF): MAF ≤ 0.1, 0.1 < MAF ≤ 0.2, 0.2 < MAF ≤ 0.3, 0.3 < MAF ≤ 0.4, and MAF > 0.4. These categories account for the fact that SNPs with higher MAF have a larger range of

possible allele frequencies differences between the two parent populations. We then calculated a diapause-associated *p*-value as the percentile of the distribution of absolute value of allele frequency difference (|AFD|) for each SNP within each category.

We calculated diapause-associated *p*-values separately for each of the bulks. In most cases (87%), we had genotyped both of the two founding parents of the bulk line. The genotypes of the parents affect the likely degree of allele frequency differentiation in the bulks; e.g., SNPs for which the parents had two of each allele are more able to become strongly differentiated solely by chance than SNPs for which the parents have one reference allele and three alternate alleles. We therefore divided SNPs into three categories, depending on whether the parents collectively had 4 identical alleles, 2 copies of the reference and the alternate allele between them, or 1 copy of one allele and 3 of the other. Within each category, we determined the percentile of each of the SNPs on the distribution of |AFD|, and assigned a *p*-value by subtracting that percentile from 1 (so that extreme allele frequency differences would have a low *p*-value). In the case of ties in |AFD|, all tied SNPs were assigned the lowest possible percentile, and thus the highest (i.e., least-significant) *p*-value. For those SNPs where the genotypes of the two parents had 4 identical alleles, the allele frequency difference between the high and low bulks was almost always 0 (98% of SNPs). In the exceptional cases in which the two parents were genotyped as having 4 identical alleles, but the bulks had a non-zero allele frequency difference, we assumed that this had arisen due to an erroneous genotype in either the parents or the bulks; since we lacked confidence in the genotyping of these SNPs, we set the *p*-value for these SNPs at 1. For those SNPs for which both parents had not been genotyped, we calculated a *p*-value as described above for the comparison of the TEMP and TROP samples. The overall

diapause-associated *p*-value for each SNP was calculated by multiplying the *p*-values for the three allele frequency differences described above (i.e., TEMP vs. TROP, high-diapause vs. low-diapause bulks for both BSA lines).

We applied three false discovery thresholds to take into account testing of multiple SNPs. In the first, only those SNPs were included for which their *p*-value was less than 0.05 / 46,736 = $1.1*10^{-6}$. We used 46,736 as the denominator in this calculation because that is the number of SNPs that meet the criteria that the three allele frequency differences (AFDs) had the same sign (i.e., TEMP vs. TROP, high-diapause vs. low-diapause bulks for both BSA lines). The other two *p*-value cutoffs were 1/46,736 = $2.1*10^{-5}$ and 5/46,736 = $1.1*10^{-4}$. These SNP sets are likely to include approximately 0.05, 1 and 5 falsely-discovered SNPs, respectively, but since the three threshold categories included approximately 4, 77 and 260 SNPs, the likelihood an any particularly SNP being a false-positive is low

Table S1: Averaged sequencing results for RNA libraries used in this study.

Libraries	Millions of total read pairs sequenced, average (range)	Filtered read pairs aligned to genome (range)	Filtered SNPs called[1]
2 F_0 parents	23 (20-25)	8 (7-8)	111,328
70 F_7 offspring	28 (20-60)	11 (5-19)	111,328
11 F_0 TEMP females	22 (17-27)	11 (7-14)	988,325
9 F_0 TROP males	16 (11-21)	7 (4-11)	988,325
High bulk, line 1	206	79	3,391,066
Low bulk, line 1	177	70	3,391,066
High bulk, line 2	208	101	3,410,627
Low bulk, line 2	181	89	3,410,627

[1] Only SNPs that were found in both F_0 parent and F_7 offspring, both TROP and TEMP populations, or in both bulks of a line, were included, which is why the same number of SNPs were found in each of those pairs.

Table S2. Individual library sequencing results for RNA libraries used in this study.

Individual Library ID	Group	Read pairs (millions)	Filtered read pairs aligned to genome (millions)
F7001	Linkage: F7 offspring	24.2	9.9
F7004	Linkage: F7 offspring	27.3	13.9
F7005	Linkage: F7 offspring	32.0	16.1
F7006	Linkage: F7 offspring	26.5	13.3
F7007	Linkage: F7 offspring	25.5	11.2
F7009	Linkage: F7 offspring	27.1	9.4
F7010	Linkage: F7 offspring	26.2	10.2
F7011	Linkage: F7 offspring	30.7	15.1
F7012	Linkage: F7 offspring	25.1	12.3
F7013	Linkage: F7 offspring	25.1	11.9
F7015	Linkage: F7 offspring	28.5	14.4
F7016	Linkage: F7 offspring	29.2	8.1
F7018	Linkage: F7 offspring	29.3	12.7
F7019	Linkage: F7 offspring	30.1	13.5
F7020	Linkage: F7 offspring	30.7	12.7
F7022	Linkage: F7 offspring	27.0	10.5
F7023	Linkage: F7 offspring	27.8	12.3
F7024	Linkage: F7 offspring	26.4	13.2
F7025	Linkage: F7 offspring	28.4	14.0
F7026	Linkage: F7 offspring	27.7	13.6
F7027	Linkage: F7 offspring	26.8	11.7
F7028	Linkage: F7 offspring	27.3	10.1
F7029	Linkage: F7 offspring	25.2	10.7
F7030	Linkage: F7 offspring	26.0	12.6
F7032	Linkage: F7 offspring	30.4	11.9
F7033	Linkage: F7 offspring	30.8	10.3
F7034	Linkage: F7 offspring	26.0	12.9
F7035	Linkage: F7 offspring	27.3	11.7
F7036	Linkage: F7 offspring	22.9	10.8
F7037	Linkage: F7 offspring	28.8	12.5
F7038	Linkage: F7 offspring	27.2	7.5
F7041	Linkage: F7 offspring	24.9	12.9
F7042	Linkage: F7 offspring	29.3	12.9
F7043	Linkage: F7 offspring	26.8	7.5
F7044	Linkage: F7 offspring	25.6	13.0
F7045	Linkage: F7 offspring	28.7	8.6
F7046	Linkage: F7 offspring	31.5	5.1
F7047	Linkage: F7 offspring	28.5	13.3
F7048	Linkage: F7 offspring	31.5	12.7
F7049	Linkage: F7 offspring	30.2	4.7
F7051	Linkage: F7 offspring	35.5	13.5
F7054	Linkage: F7 offspring	35.4	16.6
F7055	Linkage: F7 offspring	30.5	13.1
F7057	Linkage: F7 offspring	22.2	9.2
F7058	Linkage: F7 offspring	28.8	10.8
F7060	Linkage: F7 offspring	24.1	11.3
F7064	Linkage: F7 offspring	32.2	8.2

F7066	Linkage: F7 offspring	23.6	12.2
F7067	Linkage: F7 offspring	20.8	10.6
F7068	Linkage: F7 offspring	26.0	13.3
F7069	Linkage: F7 offspring	26.3	12.7
F7073	Linkage: F7 offspring	26.6	8.1
F7076	Linkage: F7 offspring	27.5	13.6
F7077	Linkage: F7 offspring	28.6	14.8
F7078	Linkage: F7 offspring	23.4	8.2
F7079	Linkage: F7 offspring	26.2	12.3
F7080[1]	Linkage: F7 offspring	12.3<br>19.3	10.7
F7083[1]	Linkage: F7 offspring	12.6<br>19.3	10.7
F7084[1]	Linkage: F7 offspring	11.2<br>16.6	12.3
F7086	Linkage: F7 offspring	28.8	13.9
F7087	Linkage: F7 offspring	32.8	16.8
F7093	Linkage: F7 offspring	35.5	16.5
F7094	Linkage: F7 offspring	35.6	14.0
F7096	Linkage: F7 offspring	24.7	12.2
F7097	Linkage: F7 offspring	22.2	10.4
F7098	Linkage: F7 offspring	23.2	6.2
F7102	Linkage: F7 offspring	19.8	8.7
F7103	Linkage: F7 offspring	60.1	19.2
F7106	Linkage: F7 offspring	36.7	13.1
F7108	Linkage: F7 offspring	35.4	17.7
BH2	BSA: high bulk, line 1	206	79
BL2	BSA: low bulk, line 1	177	70
BH3	BSA: high bulk, line 2	208	101
BL3	BSA: low bulk, line 2	181	89
M01	BSA: temperate population	17.3	9.3
M02	BSA: temperate population	17.2	9.1
M03	BSA: temperate population	18.5	6.9
M04	BSA: temperate population	19.3	10.0
M05	BSA: temperate population	22.6	10.8
M06[2]	BSA: temperate population	20.4	10.7
M07[3]	Linkage: F_0 parent<br>BSA: temperate population	25.3	8.4<br>11.3
M08	BSA: temperate population	27.1	12.9
M09	BSA: temperate population	24.2	12.7
M10	BSA: temperate population	27.3	9.0
M16	BSA: temperate population	27.1	13.8
K01	BSA: tropical population	13.4	7.1
K02	BSA: tropical population	15.3	7.0
K03	BSA: tropical population	11.1	5.1
K04	BSA: tropical population	16.9	8.2
K05[2]	BSA: tropical population	21.1	10.9
K06[3]	Linkage: F_0 parent<br>BSA: tropical population	19.7	6.9<br>9.0
K07	BSA: tropical population	13.3	4.0
K08	BSA: tropical population	10.6	3.5
K16	BSA: tropical population	20.9	9.5

[1] These libraries were sequenced on two different flowcells and later combined.

[2] These individuals were the founding parents of the line that produced bulk line 1.
[3] These individuals were the founding parents of the line that produced bulk line 2 and the individuals used for linkage mapping.

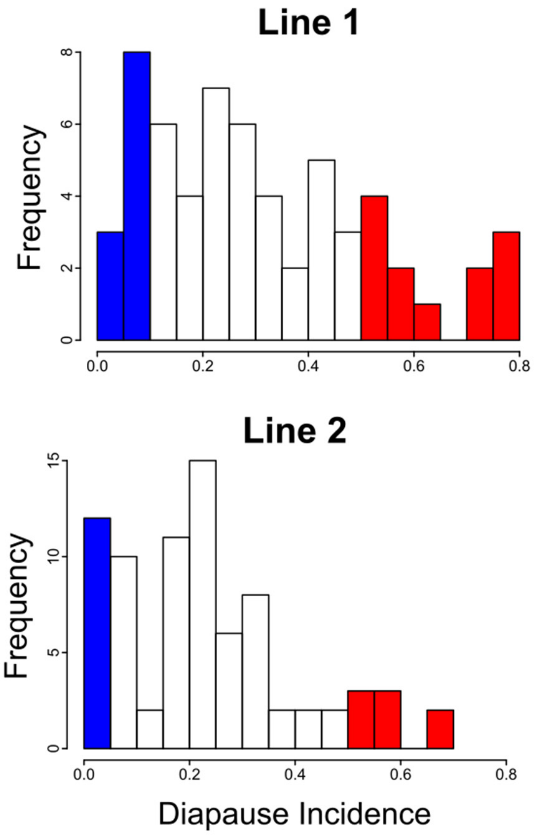

Figure S1: Diapause incidence of intercross F_4 females in two independant bulk-segregant lines. Blue bars show females chosen for low-diapause bulks within each line; red bars show females chosen for high-diapause bulks.

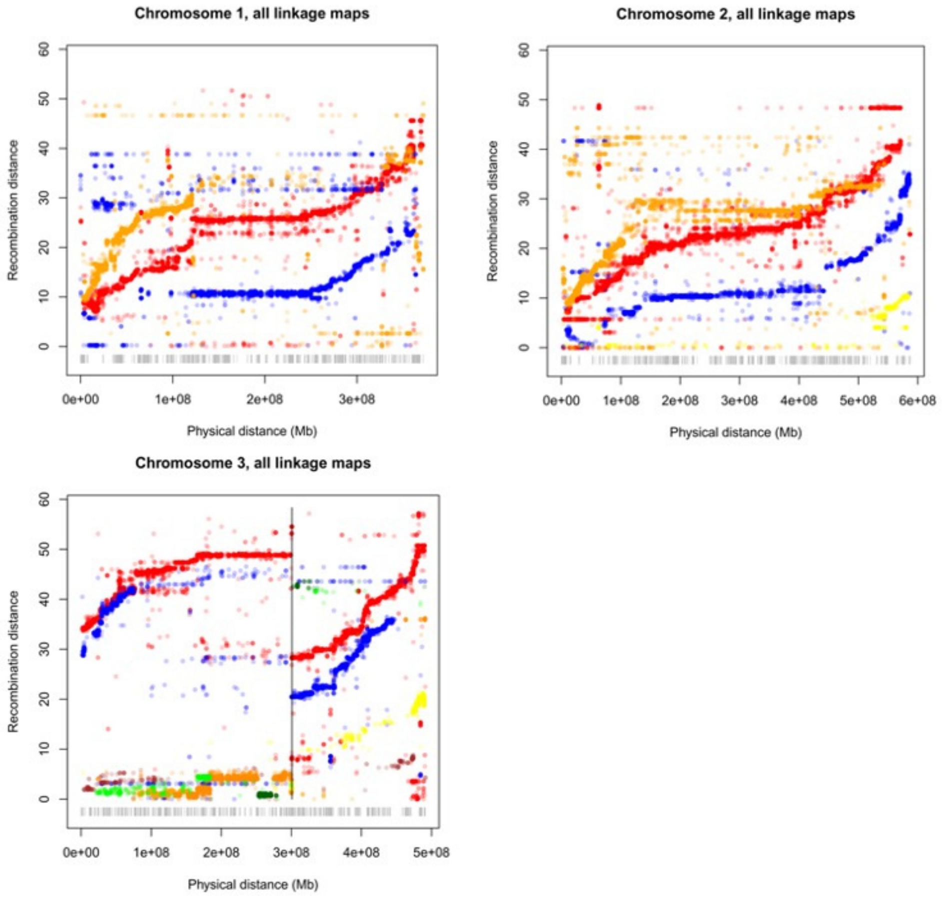

Figure S2: Linkage maps for the three *Ae. albopictus* chromosomes. Each point shows the physical (x-axis) and recombination (y-axis) distance of a single SNP marker from one end of the linkage group. The gray ticks at the bottom show the boundaries between scaffolds. Red points show the linkage map based on those markers for which both F_0 parents were homozygous for different alleles. Blue and orange points show the linkage maps based on those markers for which F_0 parents had AB female x AA male and AA female x AB male genotypes, respectively. For Chromosome 2, the AA x AB linkage map was split into two (orange and yellow points). For Chromosome 3, the AA x AB linkage map was split into six sub-maps (the various non-red and non-blue colors). The vertical bar on the Chromosome 3 linkage map shows where we split and inverted the anterior arm of the chromosome based on the results of the synteny comparison with *Ae. aegypti.*

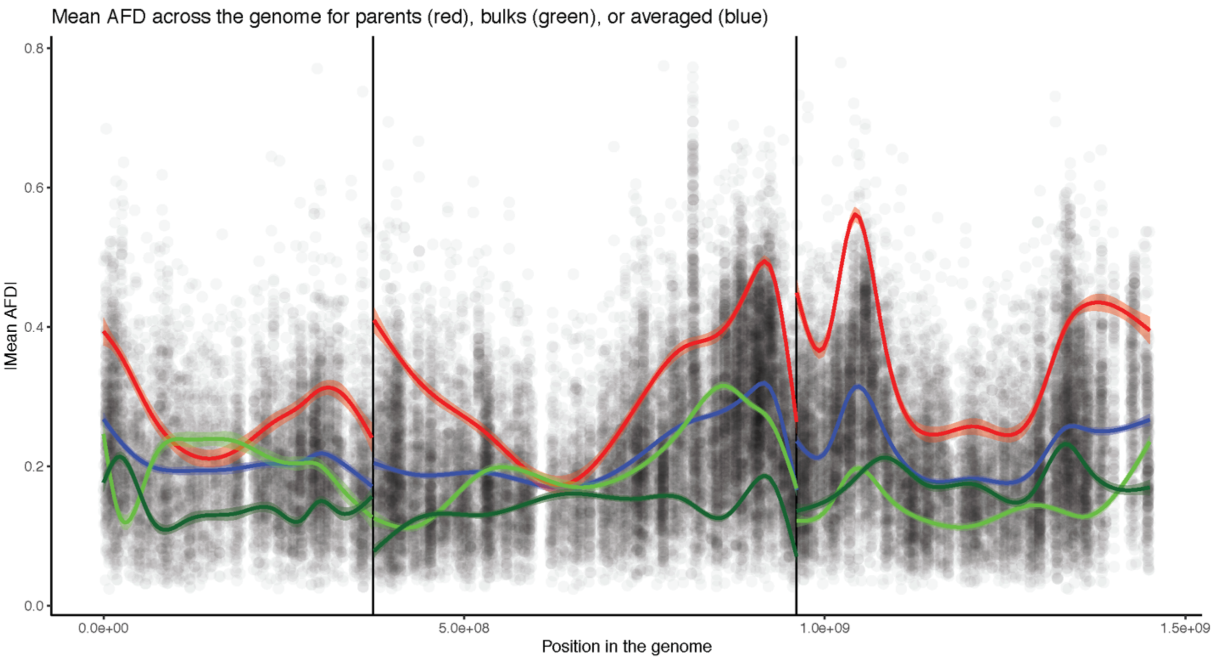

Figure S3: Regions of elevated allele frequency difference are driven by the comparison between natural populations. Axes, gray dots and blue lines as in Figure 4 from the main text. Red line shows the trend for the TEMP vs. TROP |AFD|, green lines show the trend for the two bulks.

Table S3. Coordinates on the AalbF3 assembly of candidate diapause SNPs at false discovery thresholds (FDR-level) of 0.05, 1 and 5 as described in text.

FDR-level	Scaffold	Position
0.05	chr3.19	2261869
0.05	chr3.1	3462509
0.05	chr1.5	425695
0.05	chr2.151	9557328
1	chr2.186	15550749
1	chr2.190	1106906
1	chr2.186	29098689
1	chr3.45	11850311
1	chr3.166	1169642
1	chr3.19	2261869
1	chr1.152	5097418
1	chr1.152	5097415
1	chr1.163	581239
1	chr2.196	4832063

1	chr2.163	2465993
1	chr1.34	761479
1	chr2.186	32813627
1	chr2.181	6783
1	chr2.19	9760777
1	chr2.157	918799
1	chr2.19	10459354
1	chr2.186	17679841
1	chr2.186	17679839
1	chr2.186	31379554
1	chr2.186	4486692
1	chr2.186	3412873
1	chr2.186	29098752
1	chr2.182	7297510
1	chr3.7	538792
1	chr3.7	538783
1	chr2.181	6803
1	chr2.163	2469013
1	chr2.163	2469010
1	chr2.163	2468886
1	chr2.163	2467766
1	chr2.163	2467742
1	chr2.163	2467727
1	chr2.163	2467721
1	chr2.163	2467637
1	chr2.163	2467613
1	chr2.163	2467601
1	chr2.163	2467588
1	chr2.163	2467586
1	chr2.163	2467568
1	chr2.163	2467562
1	chr2.163	2467535
1	chr2.163	2466653
1	chr2.163	2466593
1	chr2.163	2466418
1	chr3.136	1364946
1	chr3.166	3322334
1	chr3.27	10601009
1	chr3.24	350946

1	chr3.24	350853
1	chr1.10	1304806
1	chr3.1	3462509
1	chr3.136	1764215
1	chr3.20	7759813
1	chr1.37	409384
1	chr1.152	5883846
1	chr2.190	1106737
1	chr2.205	4214520
1	chr2.186	25933766
1	chr2.182	2721664
1	chr2.196	1706310
1	chr1.5	425695
1	chr2.186	25425063
1	chr2.196	437179
1	chr2.123	2652146
1	chr3.27	2456597
1	chr3.25	80308
1	chr2.163	4202864
1	chr2.163	2706299
1	chr3.147	105793
1	chr1.37	409338
1	chr2.151	9557328
1	chr2.163	5560527
1	chr3.1	1143798
1	chr3.40	7840460
1	chr3.27	10600965
1	chr1.100	64767
5	chr2.196	3223463
5	chr2.196	3223454
5	chr2.193	272110
5	chr2.186	15550749
5	chr2.186	1019659
5	chr2.186	27209638
5	chr2.186	27209611
5	chr2.179	3259424
5	chr2.179	3259013
5	chr2.155	968930
5	chr2.155	968888

5	chr2.140	5745049
5	chr3.178	2762556
5	chr3.178	2763121
5	chr3.178	2763160
5	chr3.9	5934379
5	chr2.196	3537815
5	chr2.196	15028517
5	chr2.190	1106906
5	chr2.186	1019654
5	chr2.186	29098689
5	chr2.186	24446432
5	chr2.163	1729465
5	chr2.196	3675128
5	chr2.186	794300
5	chr2.186	794294
5	chr3.45	11850311
5	chr3.29	6391766
5	chr2.140	5066312
5	chr2.196	5213745
5	chr2.172	3659293
5	chr2.163	5791823
5	chr3.139	2487053
5	chr3.139	2487054
5	chr3.166	1169642
5	chr3.197	47646
5	chr1.7	4496272
5	chr1.7	11342339
5	chr2.196	2173800
5	chr2.196	2173778
5	chr2.196	2173756
5	chr2.182	3721221
5	chr2.182	2410438
5	chr3.19	2261869
5	chr1.133	1996330
5	chr1.152	5097418
5	chr1.152	5097415
5	chr1.163	581239
5	chr1.90	16885091
5	chr2.196	4832063

5	chr2.179	4209591
5	chr3.28	1069858
5	chr2.196	9349925
5	chr3.166	4187577
5	chr2.198	2955602
5	chr2.186	21872396
5	chr2.186	21872393
5	chr2.140	5270817
5	chr3.77	12542171
5	chr2.196	437294
5	chr2.196	437284
5	chr2.163	543064
5	chr2.163	2465993
5	chr2.163	2465948
5	chr3.139	2392329
5	chr3.39	1285018
5	chr1.34	761479
5	chr1.7	8514363
5	chr1.7	5266097
5	chr1.7	5266096
5	chr1.7	5266021
5	chr1.133	4125024
5	chr2.17	23676261
5	chr2.17	23676260
5	chr2.186	32813627
5	chr2.181	6783
5	chr1.10	1304770
5	chr2.190	1211687
5	chr2.190	320798
5	chr2.19	9760777
5	chr2.182	7077973
5	chr2.182	7077960
5	chr2.157	918799
5	chr3.127	529064
5	chr3.136	7127207
5	chr2.19	10459354
5	chr2.17	23998060
5	chr2.17	23676579
5	chr2.186	17679841

5	chr2.186	17679839
5	chr2.186	31379569
5	chr2.186	31379554
5	chr2.186	4486692
5	chr2.186	3412873
5	chr2.186	1067749
5	chr2.186	29608815
5	chr2.186	29098752
5	chr2.182	7144674
5	chr2.182	7144648
5	chr2.182	7144645
5	chr2.182	7144627
5	chr2.182	7297520
5	chr2.182	7297510
5	chr2.175	5401222
5	chr2.165	909375
5	chr2.163	2333832
5	chr2.155	11263706
5	chr2.140	5270838
5	chr3.142	1345932
5	chr3.142	3017006
5	chr3.151	6543652
5	chr3.40	400831
5	chr3.40	400797
5	chr3.16	15877891
5	chr3.7	538792
5	chr3.7	538783
5	chr1.10	364309
5	chr2.196	129649
5	chr2.186	7938565
5	chr2.186	1322048
5	chr2.186	24411777
5	chr2.181	6803
5	chr2.177	773940
5	chr2.177	773910
5	chr2.163	3115287
5	chr2.163	2469745
5	chr2.163	2469679
5	chr2.163	2469094

5	chr2.163	2469027
5	chr2.163	2469013
5	chr2.163	2469010
5	chr2.163	2468980
5	chr2.163	2468944
5	chr2.163	2468941
5	chr2.163	2468886
5	chr2.163	2468875
5	chr2.163	2468860
5	chr2.163	2468785
5	chr2.163	2468773
5	chr2.163	2468027
5	chr2.163	2468024
5	chr2.163	2467994
5	chr2.163	2467949
5	chr2.163	2467766
5	chr2.163	2467742
5	chr2.163	2467727
5	chr2.163	2467721
5	chr2.163	2467637
5	chr2.163	2467613
5	chr2.163	2467601
5	chr2.163	2467588
5	chr2.163	2467586
5	chr2.163	2467568
5	chr2.163	2467562
5	chr2.163	2467535
5	chr2.163	2466659
5	chr2.163	2466653
5	chr2.163	2466593
5	chr2.163	2466497
5	chr2.163	2466485
5	chr2.163	2466460
5	chr2.163	2466452
5	chr2.163	2466418
5	chr2.163	2466413
5	chr2.163	2466340
5	chr2.163	2466338
5	chr2.163	2465642

5	chr2.140	5066272
5	chr3.136	1364946
5	chr3.145	355852
5	chr3.145	355896
5	chr3.166	3322334
5	chr3.27	10898419
5	chr3.27	10601009
5	chr3.27	10348713
5	chr3.25	2426063
5	chr3.24	350946
5	chr3.24	350853
5	chr1.10	364332
5	chr1.106	3958727
5	chr3.20	9911836
5	chr1.10	1304806
5	chr2.194	122494
5	chr2.175	8260158
5	chr2.170	2952640
5	chr2.170	2952631
5	chr2.170	2952630
5	chr2.140	12404709
5	chr3.1	3462509
5	chr3.136	1764215
5	chr3.20	3798173
5	chr3.20	7759813
5	chr1.37	409384
5	chr1.10	1319699
5	chr2.186	1341880
5	chr2.163	2874203
5	chr3.142	1349792
5	chr3.142	1349795
5	chr3.142	1349803
5	chr3.145	115123
5	chr3.145	117534
5	chr3.16	15823971
5	chr1.152	5883846
5	chr2.190	1106737
5	chr2.205	8869318
5	chr2.205	4214520

5	chr2.186	29848220
5	chr2.186	25933766
5	chr2.182	2721664
5	chr3.166	7908016
5	chr3.28	1285853
5	chr1.7	19082195
5	chr2.196	1706310
5	chr3.142	4044665
5	chr3.169	3826
5	chr3.16	1099913
5	chr1.170	660409
5	chr1.5	425695
5	chr2.196	3899105
5	chr2.186	27735884
5	chr2.163	2686028
5	chr3.142	2512406
5	chr3.27	2456642
5	chr3.142	4038468
5	chr3.61	157367
5	chr2.196	3231168
5	chr2.49	432784
5	chr2.186	29848218
5	chr2.186	25425063
5	chr2.165	909384
5	chr2.163	6566258
5	chr2.196	437179
5	chr2.175	3744730
5	chr2.123	2652146
5	chr3.122	5058945
5	chr3.27	2456604
5	chr3.27	2456597
5	chr3.27	2324689
5	chr3.25	80308
5	chr2.163	4202864
5	chr2.163	2706299
5	chr3.147	105793
5	chr3.147	858369
5	chr1.50	9046252
5	chr1.37	409338

5	chr1.139	6208060
5	chr2.71	302561
5	chr2.151	9557328
5	chr3.142	655274
5	chr3.61	775955
5	chr1.161	257707
5	chr2.186	15861726
5	chr2.166	55553
5	chr2.163	5560527
5	chr3.1	1143798
5	chr3.147	672511
5	chr3.40	7840460
5	chr3.27	10600965
5	chr3.27	2257250
5	chr1.100	64767

Table S4. Genes located within 50Kb of candidate diapause SNPs at false discovery thresholds (FDR-level) of 0.05, 1 and 5 as described in text.

FDR-level	scaffold	gene_name	gene_id
0.05	chr1.5	gametogenetin-binding protein 2-like	LOC109402174
0.05	chr1.5	segmentation polarity homeobox protein engrailed-like	LOC109402178
0.05	chr1.5	casein kinase II subunit alpha	LOC109413239
0.05	chr3.1	homeobox protein OTX2-A	LOC109411232
0.05	chr3.19	ice-structuring glycoprotein	LOC109398746
0.05	chr3.19	3-ketodihydrosphingosine reductase	LOC109398749
0.05	chr3.19	methyl-CpG-binding domain protein 3%2C	LOC109398750
0.05	chr3.19	vacuolar protein sorting-associated protein 45	LOC109398753
0.05	chr3.19	nuclear pore complex protein Nup58	LOC109398754
0.05	chr3.19	metaxin-1 homolog	LOC109398756
0.05	chr3.19	uncharacterized LOC109424986	LOC109424986
1	chr1.10	molybdenum cofactor sulfurase 3	LOC109415232
1	chr1.10	26S proteasome regulatory subunit 8	LOC109415235
1	chr1.10	pyrimidine-specific ribonucleoside hydrolase RihA-like	LOC109415237
1	chr1.10	protein artemis-like	LOC109428353
1	chr1.10	mannosyl-oligosaccharide alpha-1%2C2-mannosidase IA-like%2C	LOC109428360
1	chr1.10	migration and invasion enhancer 1-like	LOC115256449
1	chr1.10	activating signal cointegrator 1-like	LOC115256586

1	chr1.152	uncharacterized LOC109403881	LOC109403881
1	chr1.152	uncharacterized LOC109403974	LOC109403974
1	chr1.152	condensin complex subunit 1-like%2C	LOC109404013
1	chr1.152	pinin-like%2C	LOC109413333
1	chr1.152	uncharacterized LOC109413349	LOC109413349
1	chr1.152	sorting nexin-12-like	LOC109432030
1	chr1.152	uncharacterized LOC109432033	LOC109432033
1	chr1.152	band 4.1-like protein 4B%2C	LOC109432042
1	chr1.152	actin-87E	LOC109432056
1	chr1.152	actin-87E-like	LOC109432057
1	chr1.152	actin-like	LOC109432070
1	chr1.152	uncharacterized LOC115254117	LOC115254117
1	chr1.163	protein scarlet-like	LOC109422176
1	chr1.163	uncharacterized LOC115260019	LOC115260019
1	chr1.34	uncharacterized LOC109401916%2C	LOC109401916
1	chr1.34	protein kibra	LOC109414692
1	chr1.37	brefeldin A-inhibited guanine nucleotide-exchange protein 3-like	LOC109432078
1	chr1.37	fatty acid hydroxylase domain-containing protein 2-like;start_range=.,9498	LOC115258418
1	chr1.37	malignant T-cell-amplified sequence 1 homolog	LOC115269045
1	chr1.5	gametogenetin-binding protein 2-like	LOC109402174
1	chr1.5	segmentation polarity homeobox protein engrailed-like	LOC109402178
1	chr1.5	casein kinase II subunit alpha	LOC109413239
1	chr2.123	facilitated trehalose transporter Tret1-like	LOC109404663
1	chr2.123	facilitated trehalose transporter Tret1-like	LOC109433068
1	chr2.123	facilitated trehalose transporter Tret1	LOC109433069
1	chr2.157	proteasome subunit alpha type-1-like	LOC109429097
1	chr2.157	mediator of RNA polymerase II transcription subunit 16-like	LOC115258794
1	chr2.163	translation factor GUF1 homolog%2C mitochondrial	LOC109414223
1	chr2.163	collagen alpha-1(IV) chain%2C	LOC109417703
1	chr2.163	SPRY domain-containing SOCS box protein 3%2C	LOC109417706
1	chr2.163	protein MEMO1-like	LOC109417716
1	chr2.163	leucine-rich repeat and calponin homology domain-containing protein 1%2C	LOC109417717
1	chr2.163	protein MON2 homolog	LOC109417726
1	chr2.163	vascular endothelial growth factor receptor kdr-like%2C	LOC109417728
1	chr2.163	probable palmitoyltransferase ZDHHC24	LOC115253935
1	chr2.163	suppressor APC domain-containing protein 2-like	LOC115255361
1	chr2.163	uncharacterized LOC115255496	LOC115255496

1	chr2.163	COPII coat assembly protein sec16-like	LOC115255497
1	chr2.163	collagen alpha-1(IV) chain-like	LOC115255505
1	chr2.163	collagen alpha-5(IV) chain-like	LOC115255506
1	chr2.181	repressor of RNA polymerase III transcription MAF1 homolog	LOC115264869
1	chr2.182	phosphatidylinositol 4-phosphate 5-kinase type-1 alpha-like%2C	LOC109416244
1	chr2.182	protein daughterless	LOC109416248
1	chr2.182	protein phosphatase 1 regulatory subunit 42-like	LOC109416265
1	chr2.182	annulin	LOC109430162
1	chr2.182	lipase member H	LOC109430164
1	chr2.186	elongation factor G%2C mitochondrial	LOC109404794
1	chr2.186	pre-mRNA-splicing factor ATP-dependent RNA helicase DHX16-like	LOC109404796
1	chr2.186	potassium voltage-gated channel protein Shaw%2C	LOC109409153
1	chr2.186	uncharacterized LOC109410844	LOC109410844
1	chr2.186	cardioactive peptide%2C	LOC109419320
1	chr2.186	protein bowel	LOC109421565
1	chr2.186	sodium-driven chloride bicarbonate exchanger-like%2C	LOC109423877
1	chr2.186	probable cysteine desulfurase%2C mitochondrial	LOC109423882
1	chr2.186	chymotrypsin-like protease CTRL-1	LOC109621365
1	chr2.186	cGMP-dependent protein kinase%2C isozyme 1-like	LOC109621380
1	chr2.186	neither inactivation nor afterpotential protein C-like	LOC109621667
1	chr2.186	matrix metalloproteinase-15-like	LOC109622311
1	chr2.186	lysosomal alpha-mannosidase%2C	LOC109622322
1	chr2.186	chymotrypsinogen B-like	LOC109622327
1	chr2.186	uncharacterized LOC115268609	LOC115268609
1	chr2.186	suppressor protein SRP40-like%2C	LOC115268611
1	chr2.186	transcription initiation factor TFIID subunit 10-like	LOC115268623
1	chr2.190	lysosomal alpha-mannosidase-like	LOC109621385
1	chr2.190	cGMP-dependent protein kinase%2C isozyme 1-like	LOC115268590
1	chr2.190	lysosomal alpha-mannosidase-like	LOC115268591
1	chr2.196	dual specificity protein phosphatase 3%2C	LOC109402145
1	chr2.196	uncharacterized LOC109402207	LOC109402207
1	chr2.196	transcription factor hamlet-like	LOC109402239
1	chr2.196	polypeptide N-acetylgalactosaminyltransferase 3	LOC109402524
1	chr2.196	ficolin-2-like	LOC109415861
1	chr2.196	trithorax group protein osa-like%2C	LOC115253453
1	chr2.205	tubulin-specific chaperone C-like%2C	LOC109404612
1	chr2.205	ATP synthase subunit g%2C mitochondrial-like	LOC109404613
1	chr2.205	protein brunelleschi	LOC109411678
1	chr2.205	V-type proton ATPase subunit H%2C	LOC109411679

1	chr2.205	cytidine deaminase-like	LOC109411684
1	chr2.205	uncharacterized LOC115261992	LOC115261992
1	chr2.205	protein HIRA homolog%2C	LOC115262165
1	chr3.1	homeobox protein OTX2-A	LOC109411232
1	chr3.136	pancreatic triacylglycerol lipase-like	LOC109426884
1	chr3.136	phospholipase A1 VesT1.02%2C	LOC109426885
1	chr3.136	phospholipase A1 member A-like	LOC109426908
1	chr3.136	pancreatic triacylglycerol lipase	LOC109426909
1	chr3.136	pancreatic triacylglycerol lipase-like	LOC109426910
1	chr3.136	pancreatic lipase-related protein 2-like	LOC109426911
1	chr3.136	uncharacterized LOC109623319%2C	LOC109623319
1	chr3.136	protein enabled%2C	LOC109623320
1	chr3.136	uncharacterized LOC109623327%2C	LOC109623327
1	chr3.147	sterol O-acyltransferase 1%2C	LOC109414895
1	chr3.147	cell wall protein RBR3-like	LOC109414898
1	chr3.147	facilitated trehalose transporter Tret1	LOC109428885
1	chr3.166	uncharacterized LOC109398036	LOC109398036
1	chr3.166	neuropeptide Y receptor type 5	LOC109399551
1	chr3.166	sphingomyelin phosphodiesterase	LOC109399584
1	chr3.166	uncharacterized LOC115253622	LOC115253622
1	chr3.19	ice-structuring glycoprotein	LOC109398746
1	chr3.19	3-ketodihydrosphingosine reductase	LOC109398749
1	chr3.19	methyl-CpG-binding domain protein 3%2C	LOC109398750
1	chr3.19	vacuolar protein sorting-associated protein 45	LOC109398753
1	chr3.19	nuclear pore complex protein Nup58	LOC109398754
1	chr3.19	metaxin-1 homolog	LOC109398756
1	chr3.19	uncharacterized LOC109424986	LOC109424986
1	chr3.24	uncharacterized LOC115270914	LOC115270914
1	chr3.25	odorant receptor 94a-like;start_range=.,4911766	LOC109401998
1	chr3.25	ninjurin-1-like%2C	LOC109402043
1	chr3.25	axin-like%2C	LOC109413707
1	chr3.27	RNA-binding protein Musashi homolog 2-like%2C	LOC109400186
1	chr3.27	glycine receptor subunit alpha-2-like	LOC115266218
1	chr3.40	GTPase-activating Rap/Ran-GAP domain-like protein 3	LOC109433290
1	chr3.40	leucine-rich repeat-containing protein 20-like%2C	LOC109433312
1	chr3.45	uncharacterized LOC109433277	LOC109433277
1	chr3.45	lazarillo protein-like	LOC109621419
1	chr3.45	outer membrane lipoprotein Blc-like	LOC109622081
1	chr3.45	apolipoprotein D-like	LOC109622083

1	chr3.7	uncharacterized LOC109421710	LOC109421710
1	chr3.7	uncharacterized LOC109428975	LOC109428975
1	chr3.7	uncharacterized LOC115268728	LOC115268728
1	chr3.7	uncharacterized LOC115268742	LOC115268742
5	chr1.10	molybdenum cofactor sulfurase 3	LOC109415232
5	chr1.10	26S proteasome regulatory subunit 8	LOC109415235
5	chr1.10	pyrimidine-specific ribonucleoside hydrolase RihA-like	LOC109415237
5	chr1.10	protein artemis-like	LOC109428353
5	chr1.10	mannosyl-oligosaccharide alpha-1%2C2-mannosidase IA-like%2C	LOC109428360
5	chr1.10	migration and invasion enhancer 1-like	LOC115256449
5	chr1.10	activating signal cointegrator 1-like	LOC115256586
5	chr1.10	molybdenum cofactor sulfurase 2	LOC115256654
5	chr1.106	autophagy-related protein 2 homolog A-like%2C	LOC109429975
5	chr1.106	uncharacterized LOC109430003	LOC109430003
5	chr1.106	uncharacterized LOC109430695	LOC109430695
5	chr1.106	non-specific lipid-transfer protein-like 1	LOC109430696
5	chr1.106	non-specific lipid-transfer protein-like 1	LOC109430699
5	chr1.106	uncharacterized LOC109430729	LOC109430729
5	chr1.133	RPII140-upstream gene protein-like	LOC109396977
5	chr1.133	endoplasmin-like	LOC109396991
5	chr1.133	bifunctional purine biosynthesis protein PURH	LOC109412575
5	chr1.133	RPII140-upstream gene protein	LOC109412577
5	chr1.133	serine-rich adhesin for platelets-like%2C	LOC109433664
5	chr1.133	serine-rich adhesin for platelets-like	LOC115259491
5	chr1.133	endoplasmin-like	LOC115259492
5	chr1.133	uncharacterized LOC115259749	LOC115259749
5	chr1.139	polyserase-2-like	LOC109623081
5	chr1.152	uncharacterized LOC109403881	LOC109403881
5	chr1.152	uncharacterized LOC109403974	LOC109403974
5	chr1.152	condensin complex subunit 1-like%2C	LOC109404013
5	chr1.152	pinin-like%2C	LOC109413333
5	chr1.152	uncharacterized LOC109413349	LOC109413349
5	chr1.152	sorting nexin-12-like	LOC109432030
5	chr1.152	uncharacterized LOC109432033	LOC109432033
5	chr1.152	band 4.1-like protein 4B%2C	LOC109432042
5	chr1.152	actin-87E	LOC109432056
5	chr1.152	actin-87E-like	LOC109432057
5	chr1.152	actin-like	LOC109432070
5	chr1.152	uncharacterized LOC115254117	LOC115254117

5	chr1.161	protein D2	LOC109429318
5	chr1.163	protein scarlet-like	LOC109422176
5	chr1.163	uncharacterized LOC115260019	LOC115260019
5	chr1.170	protein lethal(2) giant larvae%2C	LOC109405206
5	chr1.170	fatty-acid amide hydrolase 2-like	LOC109405223
5	chr1.170	AN1-type zinc finger protein 2A	LOC109405229
5	chr1.170	uncharacterized LOC109405236	LOC109405236
5	chr1.170	uncharacterized LOC109412868	LOC109412868
5	chr1.170	exocyst complex component 2	LOC109412869
5	chr1.34	uncharacterized LOC109401916%2C	LOC109401916
5	chr1.34	protein kibra	LOC109414692
5	chr1.37	brefeldin A-inhibited guanine nucleotide-exchange protein 3-like	LOC109432078
5	chr1.37	fatty acid hydroxylase domain-containing protein 2-like;start_range=.,9498	LOC115258418
5	chr1.37	malignant T-cell-amplified sequence 1 homolog	LOC115269045
5	chr1.5	gametogenetin-binding protein 2-like	LOC109402174
5	chr1.5	segmentation polarity homeobox protein engrailed-like	LOC109402178
5	chr1.5	casein kinase II subunit alpha	LOC109413239
5	chr1.50	vesicle-associated membrane protein/synaptobrevin-binding protein	LOC109405079
5	chr1.50	vacuolar protein sorting-associated protein 4B-like%2C	LOC109416543
5	chr1.50	protein Mo25%2C	LOC109416544
5	chr1.50	vacuolar protein sorting-associated protein 4B-like	LOC109416561
5	chr1.7	uncharacterized LOC109397866	LOC109397866
5	chr1.7	disks large 1 tumor suppressor protein-like%2C	LOC109413204
5	chr1.7	probable cytosolic oligopeptidase A	LOC109413211
5	chr1.7	dynein assembly factor 4%2C axonemal-like	LOC109413212
5	chr1.7	solute carrier family 25 member 35-like%2C	LOC109413251
5	chr1.7	probable phosphorylase b kinase regulatory subunit alpha%2C	LOC109415631
5	chr1.7	phosphatidylinositide phosphatase SAC1	LOC109415652
5	chr1.7	cyclic AMP response element-binding protein B%2C	LOC109415675
5	chr1.7	potassium voltage-gated channel protein Shaker%2C	LOC109424932
5	chr1.7	protein unc-13 homolog B-like%2C	LOC109424983
5	chr1.7	uncharacterized LOC109431117	LOC109431117
5	chr1.7	T-complex protein 1 subunit beta-like	LOC115256345
5	chr1.7	p21-activated protein kinase-interacting protein 1-like	LOC115256346
5	chr1.7	uncharacterized LOC115256417	LOC115256417
5	chr1.7	paired box protein Pax-6%2C	LOC115256418
5	chr1.7	nose resistant to fluoxetine protein 6-like	LOC115256571

5	chr1.7	uncharacterized LOC115256573	LOC115256573
5	chr1.7	uncharacterized LOC115256793	LOC115256793
5	chr1.90	protein sidekick-2-like	LOC109432221
5	chr2.123	facilitated trehalose transporter Tret1-like	LOC109404663
5	chr2.123	facilitated trehalose transporter Tret1-like	LOC109433068
5	chr2.123	facilitated trehalose transporter Tret1	LOC109433069
5	chr2.140	proton-associated sugar transporter A	LOC109402171
5	chr2.140	structural maintenance of chromosomes protein 4	LOC109408120
5	chr2.140	plasminogen receptor (KT)	LOC109408121
5	chr2.140	enolase%2C	LOC109408124
5	chr2.140	uncharacterized LOC109408126%2C	LOC109408126
5	chr2.140	mite group 2 allergen Gly d 2.02-like	LOC109408133
5	chr2.140	uncharacterized LOC109408136%2C	LOC109408136
5	chr2.140	phenoloxidase-activating factor 3-like	LOC109409285
5	chr2.140	uncharacterized LOC109419020	LOC109419020
5	chr2.140	cell division control protein 1-like	LOC109419028
5	chr2.140	phenoloxidase-activating factor 3-like	LOC109419553
5	chr2.140	phenoloxidase-activating factor 3	LOC109419569
5	chr2.140	uncharacterized LOC109430388	LOC109430388
5	chr2.140	putative histone deacetylase complex subunit cti6	LOC115266898
5	chr2.140	uncharacterized LOC115266955	LOC115266955
5	chr2.155	methylcrotonoyl-CoA carboxylase subunit alpha%2C mitochondrial-like	LOC109405612
5	chr2.155	zinc finger protein 271-like	LOC109405613
5	chr2.155	MAGUK p55 subfamily member 7%2C	LOC109405615
5	chr2.155	beta-ureidopropionase	LOC109416534
5	chr2.155	rab3 GTPase-activating protein catalytic subunit-like	LOC109416535
5	chr2.157	proteasome subunit alpha type-1-like	LOC109429097
5	chr2.157	mediator of RNA polymerase II transcription subunit 16-like	LOC115258794
5	chr2.163	translation factor GUF1 homolog%2C mitochondrial	LOC109414223
5	chr2.163	uncharacterized LOC109417557	LOC109417557
5	chr2.163	glutathione S-transferase 1-like%2C	LOC109417558
5	chr2.163	glutathione S-transferase 1-like	LOC109417559
5	chr2.163	glutathione S-transferase 1-like	LOC109417694
5	chr2.163	glutathione S-transferase 1-like	LOC109417695
5	chr2.163	glutathione S-transferase 1-like	LOC109417699
5	chr2.163	collagen alpha-1(IV) chain%2C	LOC109417703
5	chr2.163	SPRY domain-containing SOCS box protein 3%2C	LOC109417706
5	chr2.163	mitogen-activated protein kinase 1-like	LOC109417712

5	chr2.163	protein MEMO1-like	LOC109417716
5	chr2.163	leucine-rich repeat and calponin homology domain-containing protein 1%2C	LOC109417717
5	chr2.163	protein MON2 homolog	LOC109417726
5	chr2.163	vascular endothelial growth factor receptor kdr-like%2C	LOC109417728
5	chr2.163	uncharacterized LOC109417730	LOC109417730
5	chr2.163	CCHC-type zinc finger protein CG3800%2C	LOC109417743
5	chr2.163	SPRY domain-containing SOCS box protein 3-like	LOC109418099
5	chr2.163	collagen alpha-5(IV) chain-like	LOC109418207
5	chr2.163	proline-rich extensin-like protein EPR1%2C	LOC109419826
5	chr2.163	receptor-type tyrosine-protein phosphatase kappa%2C	LOC109420026
5	chr2.163	E3 ubiquitin-protein ligase TRIM37-like	LOC109420030
5	chr2.163	outer dense fiber protein 3-like	LOC109425500
5	chr2.163	uncharacterized LOC109428206	LOC109428206
5	chr2.163	probable palmitoyltransferase ZDHHC24	LOC115253935
5	chr2.163	cell wall protein DAN4-like%2C	LOC115253947
5	chr2.163	neuralized-like protein 2	LOC115253948
5	chr2.163	suppressor APC domain-containing protein 2-like	LOC115255361
5	chr2.163	glutathione S-transferase 1-like	LOC115255365
5	chr2.163	glutathione S-transferase 1-like	LOC115255368
5	chr2.163	glutathione S-transferase E14-like	LOC115255371
5	chr2.163	inhibitor of Bruton tyrosine kinase-like	LOC115255378
5	chr2.163	uncharacterized LOC115255379	LOC115255379
5	chr2.163	GATOR complex protein WDR59-like%2C	LOC115255381
5	chr2.163	uncharacterized LOC115255382	LOC115255382
5	chr2.163	carcinine transporter-like	LOC115255394
5	chr2.163	probable nuclear hormone receptor HR38	LOC115255395
5	chr2.163	uncharacterized LOC115255496	LOC115255496
5	chr2.163	COPII coat assembly protein sec16-like	LOC115255497
5	chr2.163	collagen alpha-1(IV) chain-like	LOC115255505
5	chr2.163	collagen alpha-5(IV) chain-like	LOC115255506
5	chr2.165	bromodomain adjacent to zinc finger domain protein 1A-like	LOC109416656
5	chr2.165	probable serine/threonine-protein kinase tsuA	LOC115255296
5	chr2.17	protein MTO1 homolog%2C mitochondrial	LOC109423260
5	chr2.17	synaptic vesicle 2-related protein	LOC109423347
5	chr2.17	retinol dehydrogenase 12-like%2C	LOC109423348
5	chr2.17	uncharacterized LOC109423355%2C	LOC109423355
5	chr2.17	potassium voltage-gated channel subfamily H member 6%2C	LOC109423356
5	chr2.170	nephrin-like	LOC109399785

5	chr2.172	ubiquitin carboxyl-terminal hydrolase 7%2C	LOC109420687
5	chr2.175	trissin receptor%2C	LOC109419738
5	chr2.175	uncharacterized LOC109419739%2C	LOC109419739
5	chr2.175	dedicator of cytokinesis protein 9-like%2C	LOC109419769
5	chr2.175	UBX domain-containing protein 7-like%2C	LOC109420048
5	chr2.175	cyclin-dependent kinase 5 activator 1-like	LOC109420208
5	chr2.175	uncharacterized LOC115253936	LOC115253936
5	chr2.177	uncharacterized LOC115262467%2C	LOC115262467
5	chr2.177	group XV phospholipase A2-like%2C	LOC115262468
5	chr2.177	ras-related and estrogen-regulated growth inhibitor-like protein	LOC115262469
5	chr2.179	coiled-coil domain-containing protein 40	LOC109430129
5	chr2.179	zinc finger protein 561	LOC109430130
5	chr2.179	alpha-amylase 3-like	LOC109430131
5	chr2.179	monocarboxylate transporter 7-like	LOC109430179
5	chr2.179	calmodulin-lysine N-methyltransferase-like%2C	LOC109430182
5	chr2.179	protein expanded	LOC109433586
5	chr2.179	mitochondrial basic amino acids transporter%2C	LOC109433587
5	chr2.181	repressor of RNA polymerase III transcription MAF1 homolog	LOC115264869
5	chr2.182	anoctamin-10%2C	LOC109405621
5	chr2.182	phosphatidylinositol 4-phosphate 5-kinase type-1 alpha-like%2C	LOC109416244
5	chr2.182	protein daughterless	LOC109416248
5	chr2.182	protein phosphatase 1 regulatory subunit 42-like	LOC109416265
5	chr2.182	protein quiver%2C	LOC109427566
5	chr2.182	uncharacterized LOC109430153	LOC109430153
5	chr2.182	serine/threonine-protein phosphatase PP2A 65 kDa regulatory subunit%2C	LOC109430154
5	chr2.182	annulin	LOC109430162
5	chr2.182	lipase member H	LOC109430164
5	chr2.182	cyclin-H	LOC109432628
5	chr2.182	uncharacterized LOC109432638%2C	LOC109432638
5	chr2.182	lipase member H-A-like%2C	LOC115253443
5	chr2.186	elongation factor G%2C mitochondrial	LOC109404794
5	chr2.186	pre-mRNA-splicing factor ATP-dependent RNA helicase DHX16-like	LOC109404796
5	chr2.186	potassium voltage-gated channel protein Shaw%2C	LOC109409153
5	chr2.186	1%2C5-anhydro-D-fructose reductase-like	LOC109409173
5	chr2.186	uncharacterized LOC109410844	LOC109410844
5	chr2.186	uncharacterized LOC109418112	LOC109418112
5	chr2.186	transmembrane protein 189%2C	LOC109418121
5	chr2.186	uncharacterized LOC109418136	LOC109418136

5	chr2.186	tripartite motif-containing protein 45	LOC109418152
5	chr2.186	odorant receptor 30a-like	LOC109418186
5	chr2.186	uncharacterized LOC109418214	LOC109418214
5	chr2.186	cardioactive peptide%2C	LOC109419320
5	chr2.186	WW domain-binding protein 11-like	LOC109421430
5	chr2.186	protein bowel	LOC109421565
5	chr2.186	sodium-driven chloride bicarbonate exchanger-like%2C	LOC109423877
5	chr2.186	probable cysteine desulfurase%2C mitochondrial	LOC109423882
5	chr2.186	adenylate kinase isoenzyme 1-like;start_range=.,157154773	LOC109423938
5	chr2.186	uncharacterized LOC109430993	LOC109430993
5	chr2.186	peptidyl-tRNA hydrolase ICT1%2C mitochondrial-like	LOC109430997
5	chr2.186	chymotrypsin-like protease CTRL-1	LOC109621365
5	chr2.186	cGMP-dependent protein kinase%2C isozyme 1-like	LOC109621380
5	chr2.186	neither inactivation nor afterpotential protein C-like	LOC109621667
5	chr2.186	matrix metalloproteinase-15-like	LOC109622311
5	chr2.186	lysosomal alpha-mannosidase%2C	LOC109622322
5	chr2.186	chymotrypsinogen B-like	LOC109622327
5	chr2.186	tetratricopeptide repeat protein 19 homolog%2C mitochondrial-like	LOC115265561
5	chr2.186	uncharacterized LOC115265562	LOC115265562
5	chr2.186	protein abrupt-like	LOC115265563
5	chr2.186	tctex1 domain-containing protein 2-like	LOC115265630
5	chr2.186	alpha-tocopherol transfer protein-like%2C	LOC115265631
5	chr2.186	alpha-tocopherol transfer protein-like	LOC115265632
5	chr2.186	uncharacterized LOC115265633	LOC115265633
5	chr2.186	eukaryotic peptide chain release factor GTP-binding subunit ERF3A-like%2C	LOC115265634
5	chr2.186	ribosome biogenesis protein TSR3 homolog	LOC115265635
5	chr2.186	uncharacterized protein C1orf194-like	LOC115265636
5	chr2.186	serine/arginine-rich splicing factor 2-like%2C	LOC115265637
5	chr2.186	probable tRNA (guanine(26)-N(2))-dimethyltransferase	LOC115265638
5	chr2.186	probable 4-coumarate--CoA ligase 3	LOC115266859
5	chr2.186	uncharacterized LOC115266866	LOC115266866
5	chr2.186	uncharacterized LOC115268609	LOC115268609
5	chr2.186	suppressor protein SRP40-like%2C	LOC115268611
5	chr2.186	transcription initiation factor TFIID subunit 10-like	LOC115268623
5	chr2.190	coagulation factor IX-like	LOC109621360
5	chr2.190	elastase-1-like	LOC109621364
5	chr2.190	lysosomal alpha-mannosidase-like	LOC109621385

5	chr2.190	chymotrypsin-C-like	LOC109622333
5	chr2.190	cGMP-dependent protein kinase%2C isozyme 1-like	LOC115268590
5	chr2.190	lysosomal alpha-mannosidase-like	LOC115268591
5	chr2.190	leucine-rich repeat-containing protein 15-like	LOC115268596
5	chr2.190	rhodanese domain-containing protein CG4456-like	LOC115268597
5	chr2.190	rhodanese domain-containing protein CG4456-like	LOC115268598
5	chr2.193	multidrug resistance-associated protein 1-like	LOC109407008
5	chr2.193	uncharacterized LOC115262072	LOC115262072
5	chr2.193	multidrug resistance-associated protein 1-like	LOC115262073
5	chr2.193	multidrug resistance-associated protein 1-like	LOC115262076
5	chr2.194	uncharacterized LOC109429599	LOC109429599
5	chr2.196	dual specificity protein phosphatase 3%2C	LOC109402145
5	chr2.196	uncharacterized LOC109402207	LOC109402207
5	chr2.196	transcription factor hamlet-like	LOC109402239
5	chr2.196	NTF2-related export protein	LOC109402269
5	chr2.196	ATP-dependent DNA/RNA helicase DHX36	LOC109402297
5	chr2.196	protein Shroom	LOC109402335
5	chr2.196	gamma-butyrobetaine dioxygenase	LOC109402345
5	chr2.196	spermatogenesis-associated protein 20%2C	LOC109402466
5	chr2.196	3-phosphoinositide-dependent protein kinase 1-like	LOC109402475
5	chr2.196	piezo-type mechanosensitive ion channel component	LOC109402487
5	chr2.196	protein YIPF6-like	LOC109402498
5	chr2.196	probable phosphorylase b kinase regulatory subunit beta	LOC109402506
5	chr2.196	polypeptide N-acetylgalactosaminyltransferase 3	LOC109402524
5	chr2.196	uncharacterized protein At4g17910-like	LOC109402525
5	chr2.196	uncharacterized LOC109402726%2C	LOC109402726
5	chr2.196	multidrug resistance-associated protein 1-like	LOC109407010
5	chr2.196	transient receptor potential-gamma protein-like	LOC109407024
5	chr2.196	TGF-beta receptor type-1%2C	LOC109415848
5	chr2.196	THO complex subunit 2	LOC109415853
5	chr2.196	G-protein coupled receptor 143-like	LOC109415854
5	chr2.196	gastrulation defective protein 1 homolog	LOC109415856
5	chr2.196	uncharacterized LOC109415860	LOC109415860
5	chr2.196	ficolin-2-like	LOC109415861
5	chr2.196	nitric oxide synthase-like	LOC109416795
5	chr2.196	serine-rich adhesin for platelets	LOC109416797
5	chr2.196	AP-1 complex subunit gamma-1%2C	LOC109416800
5	chr2.196	uncharacterized LOC109429555	LOC109429555
5	chr2.196	ATP-binding cassette sub-family G member 4-like	LOC109429556

5	chr2.196	1-acyl-sn-glycerol-3-phosphate acyltransferase alpha%2C	LOC109429613
5	chr2.196	1-acyl-sn-glycerol-3-phosphate acyltransferase alpha-like	LOC109429615
5	chr2.196	multidrug resistance-associated protein 1-like	LOC109429691
5	chr2.196	trithorax group protein osa-like%2C	LOC115253453
5	chr2.196	multidrug resistance-associated protein 1-like	LOC115262119
5	chr2.196	leucine-rich repeat extensin-like protein 5	LOC115262242
5	chr2.198	UDP-glucuronosyltransferase 1-3-like%2C	LOC109429550
5	chr2.198	uncharacterized LOC109429914	LOC109429914
5	chr2.205	tubulin-specific chaperone C-like%2C	LOC109404612
5	chr2.205	ATP synthase subunit g%2C mitochondrial-like	LOC109404613
5	chr2.205	protein brunelleschi	LOC109411678
5	chr2.205	V-type proton ATPase subunit H%2C	LOC109411679
5	chr2.205	cytidine deaminase-like	LOC109411684
5	chr2.205	nesprin-1%2C	LOC109411751
5	chr2.205	sulfotransferase family cytosolic 1B member 1-like%2C	LOC109411753
5	chr2.205	dynein-1-beta heavy chain%2C flagellar inner arm I1 complex-like	LOC109420441
5	chr2.205	dynein heavy chain 2%2C axonemal	LOC109420451
5	chr2.205	uncharacterized LOC115261992	LOC115261992
5	chr2.205	protein HIRA homolog%2C	LOC115262165
5	chr2.49	uncharacterized LOC109428726	LOC109428726
5	chr2.49	putative ankyrin repeat protein RF_0381	LOC115270181
5	chr2.71	T-box transcription factor TBX6-like	LOC115263148
5	chr3.1	homeobox protein OTX2-A	LOC109411232
5	chr3.122	kinesin-like protein costa	LOC109401279
5	chr3.122	Down syndrome cell adhesion molecule-like protein Dscam2%2C	LOC115262726
5	chr3.127	nose resistant to fluoxetine protein 6-like	LOC109415352
5	chr3.127	uncharacterized LOC115268029	LOC115268029
5	chr3.136	pancreatic triacylglycerol lipase-like	LOC109426884
5	chr3.136	phospholipase A1 VesT1.02%2C	LOC109426885
5	chr3.136	phospholipase A1 member A-like	LOC109426908
5	chr3.136	pancreatic triacylglycerol lipase	LOC109426909
5	chr3.136	pancreatic triacylglycerol lipase-like	LOC109426910
5	chr3.136	pancreatic lipase-related protein 2-like	LOC109426911
5	chr3.136	uncharacterized LOC109623319%2C	LOC109623319
5	chr3.136	protein enabled%2C	LOC109623320
5	chr3.136	uncharacterized LOC109623327%2C	LOC109623327
5	chr3.136	protein bunched%2C class 2/F/G isoform-like	LOC115262674
5	chr3.139	zinc finger protein 628-like%2C	LOC109424670
5	chr3.139	peptide chain release factor 1-like%2C mitochondrial	LOC109424685

5	chr3.139	zinc finger protein 2-like%2C	LOC109424686
5	chr3.139	exosome complex component RRP41	LOC109424687
5	chr3.139	zinc finger protein 569-like	LOC109424688
5	chr3.139	zinc finger protein OZF-like%2C	LOC109424689
5	chr3.139	zinc finger protein 33A-like	LOC109424707
5	chr3.139	zinc finger protein 25-like	LOC109424708
5	chr3.139	sphingolipid delta(4)-desaturase DES1%2C	LOC109424709
5	chr3.139	ubiquitin carboxyl-terminal hydrolase	LOC109424711
5	chr3.142	RNA polymerase II elongation factor ELL2-like%2C	LOC109408615
5	chr3.142	coatomer subunit beta'-like%2C	LOC109408617
5	chr3.142	huntingtin-interacting protein 1-like%2C	LOC109408623
5	chr3.142	condensin-2 complex subunit D3	LOC109412035
5	chr3.142	zinc finger protein 2-like	LOC109412039
5	chr3.142	caspase Dronc	LOC109412040
5	chr3.142	F-box/WD repeat-containing protein 4	LOC109412041
5	chr3.142	vacuolar protein-sorting-associated protein 36%2C	LOC109412042
5	chr3.142	protein Exd1 homolog	LOC109412044
5	chr3.142	proteasomal ubiquitin receptor ADRM1 homolog%2C	LOC109412046
5	chr3.142	DNA-directed RNA polymerases I%2C II%2C and III subunit RPABC4	LOC109412048
5	chr3.142	ubiquitin-protein ligase E3C	LOC109412049
5	chr3.142	vanin-like protein 1	LOC109421936
5	chr3.142	vanin-like protein 1	LOC109421937
5	chr3.142	vanin-like protein 2%2C	LOC109421938
5	chr3.142	hsp70-Hsp90 organizing protein-like	LOC109421952
5	chr3.142	girdin-like	LOC109424641
5	chr3.142	uncharacterized LOC115262635	LOC115262635
5	chr3.142	uncharacterized LOC115262783	LOC115262783
5	chr3.145	transcription factor mef2A-like	LOC115267702
5	chr3.147	sterol O-acyltransferase 1%2C	LOC109414895
5	chr3.147	cell wall protein RBR3-like	LOC109414898
5	chr3.147	RNA-binding protein fusilli%2C	LOC109416894
5	chr3.147	liver carboxylesterase 4%2C	LOC109416898
5	chr3.147	solute carrier family 26 member 6-like%2C	LOC109416899
5	chr3.147	facilitated trehalose transporter Tret1	LOC109428885
5	chr3.151	fatty acyl-CoA reductase wat-like	LOC109397024
5	chr3.151	protein O-mannosyltransferase 1	LOC109397145
5	chr3.151	fatty acyl-CoA reductase wat-like	LOC109432268
5	chr3.151	OTU domain-containing protein 3-like	LOC115268116
5	chr3.16	odorant receptor 85c-like	LOC109405837

5	chr3.16	low-density lipoprotein receptor-like%2C	LOC109405842
5	chr3.16	ribosomal protein S6 kinase delta-1	LOC109405847
5	chr3.16	40S ribosomal protein S3	LOC109405848
5	chr3.16	developmentally-regulated GTP-binding protein 2	LOC109405853
5	chr3.16	uncharacterized LOC109405854	LOC109405854
5	chr3.16	glycine receptor subunit alpha-3%2C	LOC109405857
5	chr3.16	uncharacterized LOC109410413	LOC109410413
5	chr3.16	dual specificity protein kinase Ttk	LOC109415993
5	chr3.16	uncharacterized LOC109419222	LOC109419222
5	chr3.16	actin-histidine N-methyltransferase	LOC109419225
5	chr3.166	uncharacterized LOC109398036	LOC109398036
5	chr3.166	neuropeptide Y receptor type 5	LOC109399551
5	chr3.166	sphingomyelin phosphodiesterase	LOC109399584
5	chr3.166	1-acylglycerol-3-phosphate O-acyltransferase Pnpla3-like%2C	LOC109399662
5	chr3.166	T-related protein-like	LOC109399677
5	chr3.166	MOXD1 homolog 2-like	LOC109414400
5	chr3.166	uncharacterized LOC115253622	LOC115253622
5	chr3.169	alpha/beta hydrolase domain-containing protein 17B-like	LOC109408972
5	chr3.169	senecionine N-oxygenase-like	LOC109408978
5	chr3.169	flavin-containing monooxygenase FMO GS-OX-like 6	LOC109426475
5	chr3.169	uncharacterized LOC115253676	LOC115253676
5	chr3.178	major facilitator superfamily domain-containing protein 8-like	LOC109621446
5	chr3.178	solute carrier family 22 member 4-like	LOC109621447
5	chr3.178	major facilitator superfamily domain-containing protein 8-like	LOC109621449
5	chr3.178	feline leukemia virus subgroup C receptor-related protein 2-like	LOC109621463
5	chr3.178	major facilitator superfamily domain-containing protein 8-like	LOC115268003
5	chr3.178	major facilitator superfamily domain-containing protein 8-like	LOC115268200
5	chr3.19	ice-structuring glycoprotein	LOC109398746
5	chr3.19	3-ketodihydrosphingosine reductase	LOC109398749
5	chr3.19	methyl-CpG-binding domain protein 3%2C	LOC109398750
5	chr3.19	vacuolar protein sorting-associated protein 45	LOC109398753
5	chr3.19	nuclear pore complex protein Nup58	LOC109398754
5	chr3.19	metaxin-1 homolog	LOC109398756
5	chr3.19	uncharacterized LOC109424986	LOC109424986
5	chr3.197	Golgi apparatus protein 1-like%2C	LOC109423975
5	chr3.197	acetyl-CoA acetyltransferase%2C cytosolic-like%2C	LOC109426727
5	chr3.197	uncharacterized LOC115266166	LOC115266166
5	chr3.20	ras-related protein Rab-4B-like	LOC109413689
5	chr3.20	exocyst complex component 8	LOC109413690

5	chr3.20	ABC transporter F family member 4	LOC109413691
5	chr3.20	N-sulphoglucosamine sulphohydrolase	LOC109413692
5	chr3.20	Krueppel-like factor 3	LOC109413778
5	chr3.20	A disintegrin and metalloproteinase with thrombospondin motifs 9%2C	LOC109622416
5	chr3.20	myogenesis-regulating glycosidase-like%2C	LOC115254153
5	chr3.20	A disintegrin and metalloproteinase with thrombospondin motifs 12-like	LOC115265500
5	chr3.20	proline-rich protein 4-like	LOC115265501
5	chr3.24	uncharacterized LOC115270914	LOC115270914
5	chr3.25	odorant receptor 94a-like;start_range=.,4911766	LOC109401998
5	chr3.25	ninjurin-1-like%2C	LOC109402043
5	chr3.25	axin-like%2C	LOC109413707
5	chr3.25	glycylpeptide N-tetradecanoyltransferase-like	LOC109413885
5	chr3.25	uncharacterized LOC109413887	LOC109413887
5	chr3.27	nuclear protein localization protein 4 homolog%2C	LOC109400185
5	chr3.27	RNA-binding protein Musashi homolog 2-like%2C	LOC109400186
5	chr3.27	cytochrome P450 4c21-like	LOC109426977
5	chr3.27	probable cytochrome P450 313a4	LOC115266198
5	chr3.27	glycine receptor subunit alpha-2-like	LOC115266218
5	chr3.28	uncharacterized LOC109397518	LOC109397518
5	chr3.28	uncharacterized LOC109411644	LOC109411644
5	chr3.28	stabilizer of axonemal microtubules 1	LOC109411655
5	chr3.28	eukaryotic translation initiation factor 1A%2C X-chromosomal	LOC109411708
5	chr3.28	hexosaminidase D	LOC109621941
5	chr3.29	dual specificity protein phosphatase MPK-4-like%2C	LOC109410792
5	chr3.29	calcyclin-binding protein	LOC109410892
5	chr3.29	mitotic spindle assembly checkpoint protein MAD2B	LOC109410893
5	chr3.29	uncharacterized LOC115267549%2C	LOC115267549
5	chr3.39	DNA-dependent protein kinase catalytic subunit-like	LOC109414486
5	chr3.39	UMP-CMP kinase	LOC109414508
5	chr3.39	transmembrane protein 141	LOC109414524
5	chr3.39	conserved oligomeric Golgi complex subunit 3-like	LOC109414535
5	chr3.39	putative GTP-binding protein 6	LOC109423413
5	chr3.39	latrophilin Cirl%2C	LOC109423417
5	chr3.40	protein polybromo-1-like%2C	LOC109423385
5	chr3.40	D-amino-acid oxidase	LOC109423386
5	chr3.40	D-amino-acid oxidase-like	LOC109423387
5	chr3.40	GTPase-activating Rap/Ran-GAP domain-like protein 3	LOC109433290

5	chr3.40	leucine-rich repeat-containing protein 20-like%2C	LOC109433312
5	chr3.45	uncharacterized LOC109433277	LOC109433277
5	chr3.45	lazarillo protein-like	LOC109621419
5	chr3.45	outer membrane lipoprotein Blc-like	LOC109622081
5	chr3.45	apolipoprotein D-like	LOC109622083
5	chr3.61	uncharacterized LOC109403296%2C	LOC109403296
5	chr3.61	uncharacterized LOC109409395	LOC109409395
5	chr3.61	U4/U6 small nuclear ribonucleoprotein Prp31	LOC109431235
5	chr3.61	phosphatidylserine synthase 1%2C	LOC109431247
5	chr3.61	reactive oxygen species modulator 1-like	LOC115264609
5	chr3.7	uncharacterized LOC109421710	LOC109421710
5	chr3.7	uncharacterized LOC109428975	LOC109428975
5	chr3.7	uncharacterized LOC115268728	LOC115268728
5	chr3.7	uncharacterized LOC115268742	LOC115268742
5	chr3.77	toll-like receptor Tollo	LOC115257389
5	chr3.9	protein maelstrom homolog	LOC109418366
5	chr3.9	protein maelstrom homolog	LOC109418415
5	chr3.9	uncharacterized LOC109426145	LOC109426145

References

47. National Center for Biotechnology Information (NCBI). The UniVec Database. 2017. Available online: https://www.ncbi.nlm.nih.gov/tools/vecscreen/univec/ (accessed on 1 March 2019).

48. Bolger, A.M.; Lohse, M.; Usadel, B. A flexible trimmer for Illumina sequence data. *Bioinformatics* **2014**, *30*, 2114–2120.

49. Cox, M.P.; Peterson, D.A.; Biggs, P.J. SolexaQA: At-a-glance quality assessment of Illumina second-generation sequencing data. *BMC Bioinform.* **2010**, *11*, 485.

50. Dobin, A.; Davis, C.A.; Schlesinger, F.; Drenkow, J.; Zaleski, C.; Jha, S.; Batut, P.; Chaisson, M.; Gingeras, T.R. STAR: Ultrafast universal RNA-seq aligner. *Bioinformatics* **2013**, *29*, 15–21.

51. Broad Institute. Picard. Version 2.20.4. 2019. Available online: https://broadinstitute.github.io/picard (accessed 10 March 2020).

52. Li, H. A statistical framework for SNP calling, mutation discovery, association mapping and population genetical parameter estimation from sequencing data. *Bioinformatics* **2011**, *27*, 2987–2993.

53. McKenna, A.; Hanna, M.; Banks, E.; Sivachenko, A.; Cibulskis, K.; Kernytsky, A.; Garimella, K.; Altshuler, D.; Gabriel, S.; Daly, M.; et al. The Genome Analysis Toolkit: A MapReduce framework for analyzing next-generation DNA sequencing data. *Genome Res.* **2010**, *20*, 1297–1303.

54. Batz, Z.A.; Clemento, A.J.; Fritzenwanker, J.; Ring, T.J.; Garza, J.C.; Armbruster, P.A. Rapid adaptive evolution of the diapause program during range expansion of an invasive mosquito. *Evolution* **2020**, *74*, 1451–1465.

55.Trpiš, M. A new bleaching and decalcifying method for general use in zoology. *Can. J. Zool.* **1970**, *48*, 892–893.
